# Exogenous Tissue Inhibitor of Metalloproteinase-2 Affects Matrix Metalloproteinase-2 Expression in Conjunctival Filtering Blebs and Bleb Scarring in Rats

**DOI:** 10.1155/2018/9365950

**Published:** 2018-05-31

**Authors:** Ling Wang, Meng-ying Liu, Gang Yin, Na Li, Da-bo Wang

**Affiliations:** ^1^Department of Ophthalmology, Affiliated Hospital of Qingdao University, Qingdao, China; ^2^Department of Ophthalmology, Shandong Provincial Hospital Affiliated to Shandong University, Jinan, China; ^3^Qingdao Central Hospital, Qingdao, China

## Abstract

**Objective:**

To examine the effect of tissue inhibitor of metalloproteinase-2 (TIMP-2) on conjunctival filtering bleb scarring.

**Methods:**

A model of conjunctival filtering bleb was established whereby rats were injected with saline, blank adenoviral vector, or adenoviral vector carrying* TIMP-2* into the bleb. Filtration bleb formation and matrix metalloproteinase-2 (MMP-2) expression were examined.

**Results:**

All operated eyes formed obvious elevated blebs on day 1. In the normal saline group, empty plasmid group, and gene transfection group maintenance time of filtrating blebs was 5–14, 5–14, and 6–16 days, and average survival time was 8.24, 8.16, and 9.44 days, respectively. MMP-2 expression increased slightly in the gene transfection group at 3 and 5 days after surgery, reached a peak after 14 days, and then gradually decreased. MMP-2 expression was weakly positive in the normal conjunctival epithelium, but was hardly detected in the lamina propria. Seven days after surgery, the epithelium and lamina propria of the conjunctival filtering bleb exhibited strong positive expression in the empty plasmid group but only weak expression in the adenovirus group.

**Conclusion:**

Exogenous TIMP-2 interfered with local MMP-2 expression, delaying peak expression of MMP-2 and slowing the scarring of filtering blebs during wound healing of subconjunctival tissue.

## 1. Introduction

Filtration surgery is currently one of the major surgical approaches for the treatment of glaucoma, and the formation and maintenance of functional filtration blebs are crucial for its success. Postoperative wound healing consists of a series of complex dynamic processes. The main cause of filtration surgery failure results from postoperative fibroblast proliferation, collagen deposition, and filtration bleb scar formation in the surgical area, which can eventually block the drainage channel for aqueous humor established during the procedure [1, 2]. The widespread application of 5-fluorouracil and mitomycin in clinical practice has greatly improved the efficacy and success rate of filtration surgery for glaucoma [3, 4]. However, due to the side effects of these drugs, encapsulated trabeculectomy blebs with thin walls may be generated, thereby resulting in some potentially serious complications, such as long-term postoperative ocular hypotonia, bleb infection, leakage, and endophthalmitis [5–8]. Therefore, developing antiscarring drugs with more specific physiological functions and lower cytotoxicity is important for improving the success rate of glaucoma surgery.

Excessive proliferation and fibrosis of Tenon's fibroblasts in the scar tissue after glaucoma filtration surgery are the main causes of postoperative filtration bleb scarring [9, 10]. Matrix metalloproteinases (MMPs) produced by fibroblasts play an intrinsic role in the process of wound remodeling and contraction. Tissue inhibitors of metalloproteinases (TIMPs) are important endogenous inhibitors of MMPs and can protect normal tissue from damage caused by overexpression of MMPs. Our previous study examined the dynamic expression of MMP-2 and TIMP-2 during filtrating bleb formation and scarring in rat models of postoperative conjunctival filtration bleb scarring. We found that the balance between MMP-2 and TIMP-2 expression was closely related to filtration bleb scarring [11].

In the present study, we established a rat model of conjunctival filtrating bleb scarring, whereby the* TIMP-2* gene interfered with the expression of MMP-2, altering the balance between MMP-2 and TIMP-2 in the surgical region. This, in turn, affected the scarring process, inhibited filtrating bleb scarring, and increased the success rate of filtration surgery.

## 2. Materials and Methods

### 2.1. AD-TIMP-2

The AD-TIMP-2 is from Shanghai Jikai gene Chemical Technology Co., vector (GV314), Component order: CMV-MCS-3FLAG-SV40-EGFP, the viral titer = 1 × 1011 PFU/ml. Before the animal experiment, we detected the adenovirus by transfecting HEK293 cells; the obvious fluorescence can be observed in the cells, indicating the normal transfection of the target plasmid and the normal expression of the fluorescent labeling gene of the target plasmid. Western Blot detection showed that the size of the characteristic band near 21 KDa coincided with the target gene fusion protein, and the adenovirus expression of TIMP-2 gene was determined.

### 2.2. Generation of the Animal Model and Grouping

A total of 119 healthy male Sprague-Dawley rats were used. Five were randomly selected (random number method) as normal controls and received no treatment. The remaining 114 rats were included in the experimental group and were randomly divided into three groups: normal saline group, empty plasmid group, and gene transfection group. Each rat in the experimental group was implanted with an anterior chamber drainage device in the right eye to successfully establish the conjunctival bleb model [11, 12]: creating a limbus-based conjunctival flap, a full-thickness scleral tunnel was then created using a 29-gauge needle, which was inserted into the anterior chamber; a “bead” of fluid was visualized to confirm patency. The 10-0 nylon thread was used in suturing the conjunctiva and Tenon's capsule. After surgery, a microsyringe was inserted into the middle of the bleb from a position 1.5 mm from the edge of the bleb. Normal saline was injected into rats in the normal saline group, the blank adenoviral vector was injected into rats in the empty plasmid group, and an adenoviral vector carrying the* TIMP-2* gene was injected into rats in the gene transfection group (virus titer: 1 × 10^11^ PFU/mL). One microliter of solution was used in all rats. After the injection, the needle was retracted slowly, and obvious leakage of filtration blebs and reflux of the injected solution were monitored.

### 2.3. Postoperative

Following surgery, the presence of a bleb (elevation of the conjunctiva) was noted and the day that the bleb could no longer be distinguished was scored as the time of failure. The main outcome measures we observed were bleb morphology, changes in the anterior chamber, lens turbidity, and the general health of the animal. We also noted eyelid swelling, conjunctival hyperaemia healing, and corneal conditions. We recorded functional bleb survival time according to the Kronfeld bleb morphology and functional classification [13]: Type I: the wall of the vesicle is thin and there is no blood vessel, and the appearance is microcystic; Type II: flat, diffuse, and the limitation is pale and the wall is thicker; Type III: filtering bleb disappeared or bulge conjunctiva with hyperemia; the subconjunctival tissue developed scarring and hyperplasia on the scleral surface; more blood vessels are distributed on the blebs surface; Type IV: dome shaped localized protuberance, polycystic hyperplasia, and a compact cavity under the conjunctiva. As follows, diffused elevated blebs with or without microcysts, avascular, or slight hyperemia were recorded as functional filtering blebs. Localized elevated blebs in operation area with encapsulation or corkscrew vessels or massive hyperemia were recorded as nonfunctional filtering blebs.

### 2.4. Sample Collection and Processing

Each experimental group included 38 rats. The survival rate of blebs and MMP-2 protein expression were measured in each group at 1, 3, 5, 7, 14, 21, and 28 days after surgery. Eight rats were sacrificed on day 1 after surgery, and six were randomly selected to collect conjunctival and subconjunctival tissues from the surgical region of the right eye for western blot analysis. Of the six rats, three were randomly selected for TIMP-2 protein detection to confirm successful transfection, and three for MMP-2 protein expression measurement. From the remaining two rats, the whole right eye was collected to determine MMP-2 localization by immunofluorescence staining. At each of the remaining time points, five rats were sacrificed in each of the three experimental groups, and three rats were randomly selected to collect conjunctiva and subconjunctival tissue from the surgical region of the right eye for detection of MMP-2 by western blot analysis. From the remaining two rats, the whole right eye was collected to determine MMP-2 localization by immunofluorescence staining. In the normal control group, two rats were randomly selected, and intact right eyes were collected for immunofluorescence localization of MMP-2.

### 2.5. Measuring Expression of MMP-2 and TIMP-2 by Western Blot

The expression levels of MMP-2 and TIMP-2 were examined by western blot analysis. Tissues of interest were dissected with clean tools, snap-frozen, and kept on ice for immediate homogenization. In a tube, ~60 *μ*L of ice-cold lysis buffer per ~1 mg of tissue was added, and samples were homogenized using an electric homogenizer. The blade was rinsed twice with 200 *μ*L of lysis buffer, and the sample was kept at constant agitation for 2 h at 4°C. After centrifuging for 20 min at 12,000 rpm at 4°C, the tubes were placed on ice and the supernatant was aspirated to a fresh tube. Protein quantification of the supernatant was performed by the Bradford method. Equal amounts of total protein from each sample were separated by 10% SDS-PAGE and transferred to a PVDF membrane. After blocking with 10% nonfat dry milk in PBS containing 0.1% Tween-20, for 1 h at room temperature, membranes were probed with anti-MMP-2 (1 : 800; Santa Cruz) or anti–TIMP-2 (1 : 800; Santa Cruz) mouse monoclonal antibody, overnight at 4°C. This was followed by incubation with HRP-conjugated goat anti-mouse IgG (1 : 6000, Santa Cruz), secondary antibody for 1 h at room temperature. Blots were probed in parallel with an anti-GAPDH antibody as a loading control. Protein bands were visualized with an enhanced chemiluminescence kit (Enlight Western Blotting Reagents; Engreen Biosystems). Band intensities were quantified using Image-Pro Plus software (software serial number: 41M60032-00032, Media Cybernetics Company).

### 2.6. Localization of MMP2 Expression by Immunofluorescence Staining

After careful dissection, eyes were rinsed with PBS and embedded in OCT. Frozen sections (6 *μ*m) were cut at −20°C using a cryostat (Leica CM1950; Leica Biosystems, Germany), mounted on slides, and stored at −80°C until needed. Slices were fixed with 4% paraformaldehyde for 15 min at room temperature, washed twice with PBS containing 0.025% Tween-20 for 5 min, and blocked with 5% bovine serum albumin (BSA) and 0.5% Triton X-100 in PBS for 30 min at room temperature. Samples were incubated with primary antibodies [anti-MMP2 (ab37150, 1 : 200; Abcam)] overnight at 4°C, followed by secondary antibody incubation with goat anti-rabbit IgG (CW0103; 1 : 100, cwbio) and goat anti-mouse IgG (CW0145, 1 : 100; cwbio), both Cy3 conjugated, for 1 h at 37°C. We performed 4,6-diamidino-2-phenylindole (DAPI) staining for 5 min to identify cell nuclei (1 : 4000; China Beyotime Institute of Biotechnology). Negative controls were performed for each immunofluorescence staining experiment by skipping the incubation with the primary antibody, and all lacked staining. The sections were visualized and photographed using a fluorescence microscope (DFC480, Leica).

## 3. Statistical Analysis

All results are expressed as means ± standard error of the mean (SEM). Statistical analysis of MMP-2 expression at each time point was conducted using SPSS software version 17.0 (SPSS, Chicago, IL, USA). We used the Shapiro-Wilk test for normal data distribution and Levene's test to verify the homogeneity of variance between the groups pertaining to the survival rate of filtering blebs. MMP-2 expression was compared among groups using one-way analysis of variance (ANOVA). Results were considered significant at *p* < 0.05. The comparisons between two groups were conducted using least-significant difference.

## 4. Results

### 4.1. General Conditions and Anterior Segment Observation following Surgery

Following filtering surgeries, animals were examined for the presence of a bleb and further monitored for time of failure. A shallow anterior chamber occurred in 9 eyes, and these animals were discontinued from the experiment. Filtering surgeries were performed in the additional rats. Hyphema was observed in 7 eyes; however, they were completely self-absorbed after 2-3 days. Varying degrees of aqueous flare and presence of cells in the aqueous humor disappeared 2–4 days after surgery. Bleb leak, bleeding, infection, and cataract endophthalmitis complications did not occur.

### 4.2. Formation of Blebs

**Figure 1:**
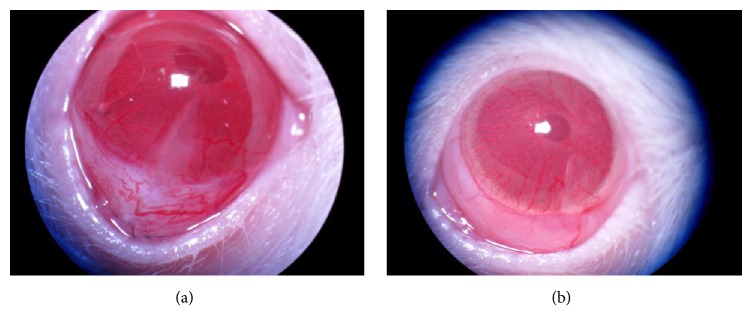
Filtration bleb morphology on day 5 after surgery. (a) Filtration blebs were flat and elevated, and vessels were observed on the surface in the empty plasmid group. (b) Filtration blebs were diffuse and elevated, and vascularization on the surface was mild with only mild conjunctival hyperemia in the gene transfection group.

On the first day after surgery, rats from all three experimental groups exhibited variously elevated filtration blebs in the operated eyes. In the normal saline group, empty plasmid group, and gene transfection group, the maintenance times of filtration blebs were 5–14, 5–14, and 6–16 days, respectively, with average survival times of 8.24, 8.16, and 9.44 days, respectively. The bleb survival at each time point in three groups is indicated in Table 1. In the saline group and empty plasmid group, vascularization was first observed on the surface of the blebs 4-5 days after surgery (Figure 1(a)). After five days, the filtration blebs in the gene transfection group remained diffuse and elevated, with a slight degree of vascularization and mild conjunctival hyperemia (Figure 1(b)).

The survival time of filtering blebs in three experimental groups was analyzed by one-way ANOVA (*F* = 3.258) and indicated significant differences between groups at each time point after surgery (*p* < 0.05). When comparing the values of two groups, no significant difference was recorded between the saline and empty plasmid groups (*p* = 0.08); however, significant differences were found between other pairings (all *p* < 0.01).

### 4.3. TIMP-2 Expression on Day 1 after Surgery

**Figure 2:**
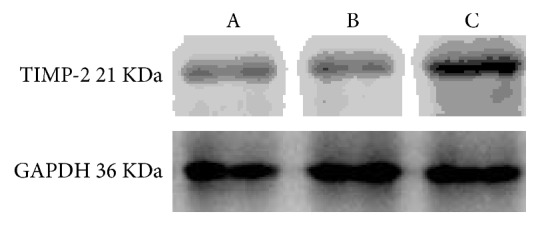
TIMP-2 expression on day 1 after surgery.* Note. *A: normal saline group; B: empty plasmid group; C: gene transfection group.

**Figure 3:**
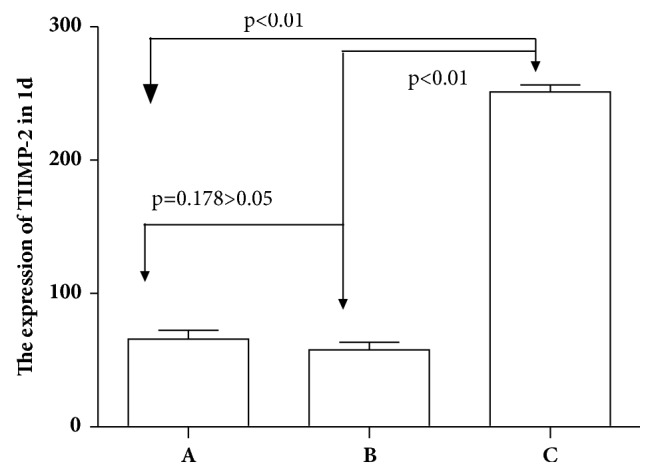
Expression of TIMP-2 expression on day 1 after surgery detected by western blot analysis (mean gray-scale value ± SEM, *n* = 3).* Note. *A: normal saline group; B: empty plasmid group; C: gene transfection group.

Next, we analyzed the expression of TIMP-2 in the conjunctival and subconjunctival tissues in rats from the three groups (Figures 2 and 3). One-way ANOVA of relative gray-scale values (*F* = 1758.324) showed significant differences between the groups (*p* < 0.01). When comparing the values between two groups at a time, no significant differences were observed between the normal saline and empty plasmid groups (*p* = 0.178); however, significant differences were found between other pairings (all *p* < 0.01). TIMP-2 expression in the gene transfection group increased on day 1 after surgery, with TIMP-2 protein levels being significantly higher than in the saline or empty plasmid groups, thus indicating successful transfection of adenovirus carrying TIMP-2.

### 4.4. Expression of MMP-2 in Conjunctival Filtration Blebs in the Three Groups at Different Time Points after Surgery

**Figure 4:**
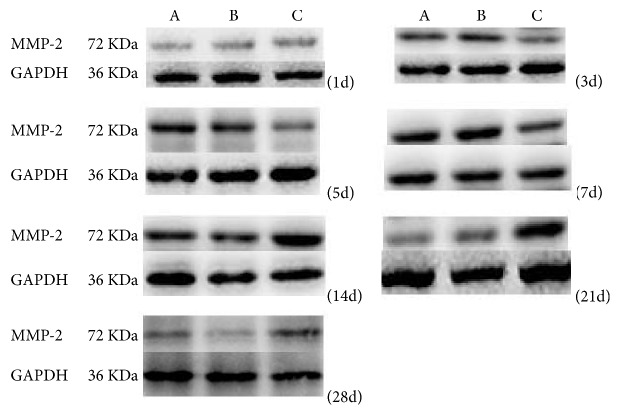
MMP-2 expression in the three experimental groups at different time points after surgery.* Note. *A: normal saline group; B: empty plasmid group; C: gene transfection group.

**Figure 5:**
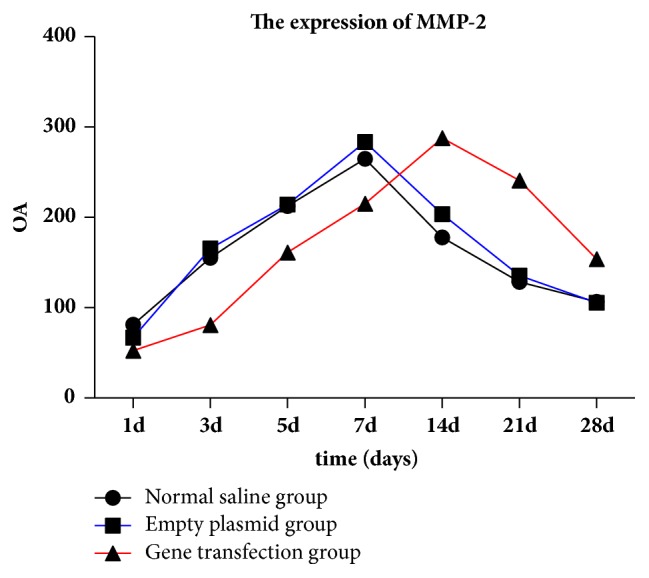
MMP-2 expression in conjunctival tissues of rats detected by western blot analysis (mean gray-scale value ± SEM, *n* = 3).

**Figure 6:**
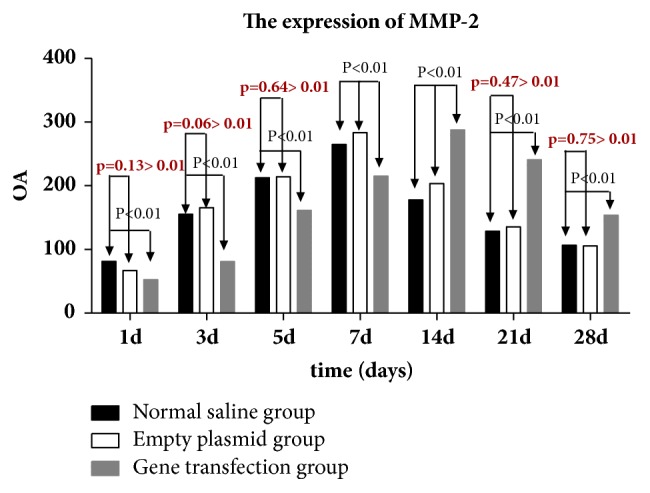
Statistical analysis of MMP-2 expression in conjunctival tissues in rats in each group.

One-way ANOVA of relative gray-scale values (*F*1d = 36.04, *F*3d = 435.5, *F*5d = 161.022, *F*7d = 133.204, *F*14d = 439.91, *F*21d = 825.35, *F*28d = 202.39; Figures 4–6) indicated significant differences between groups at each time point after surgery (*p* < 0.01). No significant difference was recorded between the saline and empty plasmid groups on day 1 (*p* = 0.13), day 3 (*p* = 0.06), day 5 (*p* = 0.64), day 21 (*p* = 0.47), or day 28 (*p* = 0.75) after surgery. Instead, significant differences were observed between all other groups at each time point (all *p* < 0.01).

Specifically, in the gene transfection group, MMP-2 expression increased slightly on days 3 and 5 and then significantly on day 7. At 14 days after surgery, the expression of MMP-2 peaked and then gradually decreased until day 28. MMP-2 expression soon after surgery was lower and the expression peak was delayed in the gene transfection group compared to that in the saline or empty plasmid groups. However, once the peak value was reached, protein levels at 21 and 28 days after surgery remained higher than in the normal saline or blank plasmid groups.

### 4.5. Expression of MMP-2 by Immunofluorescence Staining

**Figure 7:**
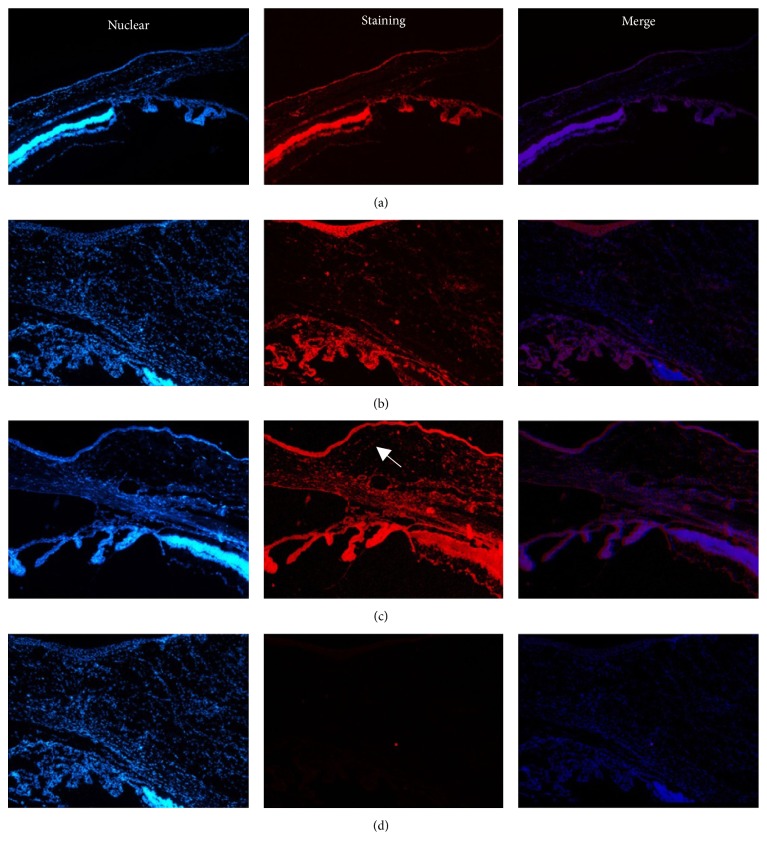
The expression of MMP-2 in the epithelial layers of the normal conjunctiva and in the conjunctival filtration area (immunofluorescence staining, 100x amplification). (a) Control group: staining of MMP-2 only in the epithelial layers of the normal conjunctiva was weak. (b) At day 7 empty plasmid group, (c) gene transfection group, and (d) negative controls (at day 7: absence of primary antibody) showed negative staining (*↗* indicates staining).

The normal conjunctival epithelium showed weak expression of MMP-2, whereas almost no expression could be detected in the lamina propria. In rats that did not undergo gene transfection, MMP-2 expression peaked on day 7 after filtration surgery, which was therefore chosen as the time point for immunofluorescence staining of MMP-2. Seven days after filtration surgery, strong expression of MMP-2 was found in the epithelium and lamina propria of the conjunctival filtrating blebs in the empty plasmid group. In contrast, in the gene transfection group, MMP-2 expression was weak in the epithelium and lamina propria of the conjunctival tissues of filtration blebs (Figure 7).

## 5. Discussion

The extracellular matrix (ECM) is a connective tissue located between cells. Normally, production and degradation of the ECM are maintained by a dynamic balance, and the ECM is involved in physiological processes, such as cell migration, differentiation, and proliferation. Under pathological conditions, the increased production and/or reduced degradation of ECM components can destroy this dynamic balance; such alterations are involved in many pathological processes, including inflammation, tumor invasion, and metastasis. MMPs are the most important class of ECM-degrading enzymes. The entire MMP family is capable of degrading almost all ECM components, except polysaccharides. MMPs are expressed in normal eye tissue. Ocular scar formation is closely related to MMP overexpression [14–17], and the imbalance between MMPs and TIMPs can cause pathological scar formation. Liu et al. [18] detected MMP-1 but not MMP3 expression in the conjunctival tissue of a rabbit model of filtration blebs. Shima et al. [10] detected expression of MMP-1 at the protein and mRNA levels by establishing a rabbit model of filtrating bleb scarring formation. Previous studies of conjunctival filtration bleb formation and scarring in rats suggested the dynamic expression of MMP-2 and TIMP-2 and the balance between these two proteins was closely related to the scarring of filtrating blebs [11]. Further studies on the specific inhibitory effect of TIMP-2 on MMP-2 may improve the success rate of filtration surgery by degrading the ECM of fibroblasts in Tenon's capsule and thus inhibiting filtration bleb scarring.

Our previous study showed that scar formation during wound repair was most critical within two weeks after surgery [11]. From days 1–3, HE staining revealed that the connective tissue of the bulbar conjunctiva was markedly oedematous and loose, with vascular dilation and containing an abundance of infiltrating neutrophils. At day 5, observation under the light microscope revealed that the oedematous connective tissue of the bulbar conjunctiva was unremarkable, that vascular dilation was alleviated, and that fibroblasts proliferated. At day 7, HE staining revealed vascular dilation and congestion with an abundance of fibroblasts, and a small amount of loose collagenous fibers around. From days 14–28, conjunctival blebs became flat and gradually disappeared. HE staining revealed collagen proliferation in the area of surgery, forming the scar. In normal rats, MMP-2 and TIMP-2 start to be expressed one day after filtration surgery and expression increases until it reaches a peak 14 days later, after which it gradually decreases. Target genes begin expression 24 hours after transfection with adenovirus. Therefore, in the present study, injection of the target gene-carrying adenovirus was programmed to interfere with the expression of MMP-2 at the time the model was generated, aiming to inhibit formation of infiltration bleb scarring. Accordingly, TIMP-2 expression increased one day after surgery, indicating a successful transfection, and protein levels became significantly higher than in the other groups.

In this study, the survival rate of filtering blebs five days after surgery was 100% in all three groups. Rats in the normal saline and empty plasmid groups started to exhibit vascularization on the surface of filtering blebs at 4-5 days after surgery, whereas, in the gene transfection group, the filtering blebs remained diffuse and elevated, with mild vascularization and slight conjunctival hyperemia. Seven days after surgery, the survival rate of filtering blebs in the gene transfection group was 70%, which was higher than in the saline and empty plasmid groups at the same time point. Some filtering blebs in the saline group showed scar formation and loss of blebs, and vascularization was prominent in the surviving blebs. In the gene transfection group, the surviving filtering blebs remained diffuse and elevated, while vascularization was not obvious, with only slight conjunctival hyperemia. Wong et al. [19] established the rabbit model of filtering blebs, and injection of ilomastat in subconjunctival tissue was used to inhibit MMPs. In the treatment group, the survival time of filtering blebs was prolonged, the filtering blebs were larger, and most of the filtering blebs were raised and diffuse. After 21 days, the filtering blebs in the control group became scarred and flat, whereas those in the treatment group were still raised and diffuse. In addition, they also exhibited fewer vessels, and the survival time of filtering blebs was longer. Accordingly, we believe that the survival time of filtering blebs was prolonged by inhibiting MMP-2 and decreasing the amount of scar tissue. The anterior chamber drainage tube was the factor which promoted the scar formation. So the mean survival time of filtering blebs was shorter in our research. This allowed for closer resemblance to clinical glaucoma implantation and represents the preferred method for characterizing progressive bleb failure. The whole process causes a typical wound healing scarring response similar to the wound healing process following glaucoma surgery in humans.

In the empty plasmid group, the expression of MMP-2 began to increase from the third day after surgery, peaked on the seventh day after surgery, and then gradually decreased. The expression of MMP-2 in the gene transfection group was not obvious on days 3 and 5 after filtration surgery, and the protein levels in the gene transfection group were lower than those in the normal saline or empty plasmid groups. We hypothesize that the injected adenovirus may induce target gene expression as early as 24 h after injection, and excessive TIMP-2 inhibits the expression of MMP-2. The expression of MMP-2 began to increase on day 7 after surgery, peaked on day 14, and then gradually decreased. These data indicate that the gene transfection group and empty plasmid group showed different MMP-2 expression trends during the scarring process, and that peak expression in the former was delayed. Early after surgery, MMP-2 levels in the gene transfection group were lower than those in the saline and blank plasmid groups, suggesting inhibition of MMP-2 expression by TIMP-2 transfection. As TIMP-2 was consumed [20], MMP-2 levels gradually increased, peaked, and then remained higher than in the other groups at 21 and 28 days after surgery. Therefore, during scarring, the expression of MMP-2 was not completely abolished but was inhibited by excessive TIMP-2. The balance between MMP-2 and TIMP-2 was disrupted, and the peak of MMP-2 expression was delayed, which in turn delayed the scarring process.

Many studies have examined the localization of MMPs and TIMPs surrounding filtering blebs. Yang et al. [21] detected the expression of MMP-2 and MMP-9 in normal conjunctival tissue. McCluskey et al. [17] used immunohistochemistry to detect the expression of MMP-1, MMP-2, and TIMP-2 around the conjunctival tissue in patients with glaucoma valve implantation. Mathalone et al. [22] reported the expression of MMP-2 and MMP-9 in conjunctival tissue, particularly the lamina propria, which is rich in cells and blood vessels, using immunohistochemistry after filtration surgery. In addition, they also observed expression of TIMP-1 and TIMP-2 in the conjunctival epithelium and lamina propria of the filtration surgical region. In this study, we used immunofluorescence staining and found only weak expression of MMP-2 in the conjunctival epithelium, with no expression in the lamina propria. However, in the empty plasmid group, strong expression was found in the epithelium and lamina propria of the conjunctival tissue on day 7 after filtration surgery, and MMP-2 expression in the gene transfection group was weak in both the epithelium and lamina propria of the conjunctival tissue in the filtering blebs. Although we did not quantify the intensity of immunofluorescence, MMP-2 expression was lower in the gene transfection group than in the blank plasmid group, consistent with the results of western blot analysis.

The study had some limitations. First, subconjunctival injection after filtration surgery is an invasive approach. Although a microsyringe was used, wound healing at the location of the needle in the conjunctiva is likely to exacerbate the entire scarring process. Second, although this study determined the effective concentration of adenovirus based on preliminary experiments, the maximum inhibitory effect of TIMP-2 was not considered; thus, optimal dose and concentration have not been established. Accordingly, interference factors should be excluded as much as possible, and the in vitro and in vivo dose-effect relationships should be established. Further studies are required to facilitate the application of MMPs and their inhibitors in scar formation after filtration surgery.

## 6. Conclusions

Here, we studied the wound healing process of subconjunctival tissue in a rat model of conjunctival filtering bleb scarring. We report that exogenous TIMP-2 interfered with local MMP-2 expression in filtering blebs, disrupting the balance between MMP-2 and TIMP-2. This delayed the peak expression of MMP-2 and slowed the scarring process in filtering blebs. Our findings may provide new insights into therapeutic approaches based on interference with scar formation after filtering operations.

## Figures and Tables

**Table 1:** Dynamic observation of survival time about filtration surgery in rats in the three groups.

Postoperative days	normal saline group			empty plasmid group			gene transfection group		
	O	E	R (%)	O	E	R (%)	O	E	R (%)
1	38	38	100	38	38	100	38	38	100
3	30	30	100	30	30	100	30	30	100
5	25	23	92	25	24	96	25	25	100
7	20	10	50	20	11	55	20	14	70
14	15	0	0	15	1	0.06	15	2	13.3
21	10	0	0	10	0	0	10	0	0
28	5	0	0	5	0	0	5	0	0

*Note. *O: cases; E: functional blebs; R: survival rates (%).
